# Supplementary material for: An ultrahigh resolution pressure sensor based on percolative metal nanoparticle arrays
Source: Nat Commun. 2019 Sep 6;10:4024. doi: 10.1038/s41467-019-12030-x (PMC6731318; doi:10.1038/s41467-019-12030-x)
Supplement: Supplementary file 1 — Supplementary Information [file 41467_2019_12030_MOESM1_ESM.pdf]

Supplementary Information

An ultrahigh resolution pressure sensor based on percolative metal nanoparticle arrays

Chen *et. al.*

Supplementary Note 1: Fabrication of sensing elements

Thin PET sheets (from about 50 to 250 μm thickness) with the smooth surface were chosen as substrates (see Supplementary Figure 1a). The PET sheets without any scar were washed with alcohol and deionized water. Silver interdigital electrodes (IDEs) were printed on the PET membranes by performing shadow mask thermal evaporation in a vacuum environment (see Supplementary Figure 1b). The IDEs contained an electrode separation of about 15 μm, with an as-prepared resistance large than 10^{10} Ω. The IDEs covered an area of several to several ten square millimeters, resulting in a huge aspect ratio (total electrode length vs. electrode separation) of the inter-electrode gaps.

Palladium (Pd) NPs were generated from a magnetron plasma gas aggregation cluster source in argon stream at a pressure of about 80 Pa and extracted to a high vacuum deposition chamber with a differential pumping system.[1, 2] And they could impact on the substrate surface with a kinetic energy up to about 1000 eV.[3] Such an impact energy is high enough to create a reactive site that pins the NP to the polymer surface to form a good particle/polymer adhesion.[4] The Pd NPs were deposited on the PET membrane covered by IDEs. The controllable physical deposition of the NPs was achieved by monitoring the conductance between the IDEs during deposition, as shown in Supplementary Figure 1c. The whole area of the IDEs was uniformly covered by the cluster-beam spot. A constant deposition rate was precisely maintained and monitored with a quartz crystal microbalance. In practice, the electric current across the IDEs gaps was measured in real-time under 1.0 V applied bias with a source meter (Keithley 2400).

A typical plot of the conductance evolution of the NP arrays, which can be described by using the percolative electron transport model, as a function of deposition time is shown in Fig. 1d. After approaching the percolation threshold, a continuous increasing of the conductance with the

deposition time, including a fine change on the rising slope of the curve near the least measurable conductance was observed. Here we demonstrate that the rising slope is positively correlated with the deposition rate of the metal NPs. Pd NP depositions with rates of 0.2, 0.25 and 0.3 $Å·s^{-1}$ were performed by controlling the discharging power at 27, 32, and 37 W in the cluster source, respectively. The main operation parameters for depositing NPs were summarized in Supplementary Table 1. Beyond the percolation threshold, the conductance of the NPs array increased with the deposition time with a gradually changed rising slope. A fine control on the rising slope with the deposition rate was shown (Supplementary Figure 2). The higher the deposition rate, the quicker the conductance increasing, resulting in a steeper rising slope.

Supplementary Note 2: Structure characterizations

To observe the scale distribution and stacking of the NP arrays, scanning transmission electron microscopy (STEM) investigation was performed using a JEOL instrument (JEM2100F) with a spherical-aberration corrector (CEOS GmbH). The images were acquired using high angle annular dark field (HAADF) detectors. Another transmission electron microscopy (TEM, FEI Tecnai F20s Twin) was used to investigate the fusion growth comparison of gold (Au), silver (Ag) and Pd NPs with lower and higher coverages. Furthermore, in order to analyze the oxidation degree of Pd NPs, the morphology of single NP was performed by the high-resolution TEM (HR-TEM).

To facilitate STEM and TEM observations, Au, Ag and Pd NPs were deposited on amorphous carbon films supported by formvar-coated copper grids.

NP arrays of gold (Au), silver (Ag) and palladium (Pd) with different coverages were prepared by cluster beam deposition. The aggregation status of the NPs in the arrays was characterized via TEM (Supplementary Figure 4). Significant spontaneous coalescence and growth among NPs

could be clearly observed in the Au and Ag NP arrays. This irreversible growth behavior will greatly influence the distribution of the nanoscale gaps in the NP arrays and induce large instability on the measured intrinsic conductance of the NP arrays. On the other hand, evidence of coalescence and growth among NPs was hard to be observed in the Pd NP arrays. Nanoscale gaps could be clearly observed between almost all the adjacent NPs. Obviously, Pd NPs were more suitable to be used to constitute percolative conducting NP arrays.

The morphologies of the Pd NP arrays on the PET membrane were examined by operating field emission scanning electron microscope as shown in Supplementary Figure 3 (FE-SEM, SU8010, Hitachi Limited).

Supplementary Figure 5a shows the XPS (X-ray photoelectron spectroscopy) spectrum of Pd NPs. The nanoparticles were deposited on a quartz substrate with a deposition rate of 0.2 $Å\cdot s^{-1}$ for 10 minutes, and placed in air environment for 30 days. The peaks of Pd 3d 5/2 and 3d 3/2 core levels are situated at the accepted binding energies for metallic Pd but contain broadened tails at the higher energy sides, which can be decomposed to additional smaller peaks corresponding to the core levels of Pd oxide, indicating that the Pd NPs are partially oxidized. The higher energy tails were eliminated after the sample surface was cleaned with Ar ion sputtering, indicating that the oxidation remained on the nanoparticle surface.

In Supplementary Figure 5b, HR-TEM image of a Pd nanoparticle is shown. Lattice images can be distinguished in the core, implying it is metallic Pd. Meanwhile, a thin amorphous shell, which is most likely PdO_x, is also distinguishable. Its thickness is measured to be about 0.5 nm on average.

Supplementary Note 3: Characterization of the response behavior of the pressure sensor

A home-made system was used to test the sensing performance of our devices as shown in Supplementary Figure 6. The pressure sensitivity of the fabricated strain sensing element was tested in a diaphragm-based piezoresistive pressure sensor configuration that was completed by mounting the PET membrane on the reference cavity using Teflon O-rings to obtain a leakproof arrangement. The electric conductance of the NP arrays on the actuation membrane was measured at different pressures with a sourcemeter (Keithley 2400) connected electrically to the IDEs through vacuum-compatible feedthroughs that were drawn outside the cavity. The integrated pressure sensor was connected to a pressure controller for applying different pressures on the sensor. A home-made set-up containing a large volume (about 7.8 litres) stainless steel chamber connected with a thin (8 mm dia.) bellows was used as the precise pressure controller. The length of the bellows could be finely adjusted with a long travel micrometer driver. The volume of the larger chamber could be flexibly modulated range from 6 mL to 7.8 L. Under static state condition, the small volume change induced a small pressure change, which could be calculated simply by employing the ideal gas model assumption. Such a system could provide a static pressure change of up to ± 40 kPa around the atmospheric pressure with an extremely high resolution of 0.1 Pa.

Supplementary Note 4: Characterization of the response of the device to strain

The strain sensing behaviors of Pd NP films were investigated by subjecting an unconstrained PET substrate to a series of bending operations which were produced by a home-made measuring apparatus as shown in Supplementary Figure 13a. The PET membrane based actuation layer was placed above the U-type holder freely, and deformed by the micrometer with an accuracy of 1 μm. The strains induced by the deformations can be expressed as follows:[5]

$$\varepsilon = \frac{T_s}{2r_b}$$

where T_s is the substrate thickness and r_b is the radius of curvature of the membrane which is bent uniformly. Considering the geometric relationship showed in Supplementary Figure 13b, r_b can be calculated from the following equation:

$$r_b^2 = (\frac{l}{2})^2 + (r_b - h)^2$$

where the value of l can be measured easily, and the value of h can be read out from the micrometer. Meanwhile, the conductance variations of the Pd NP arrays were acquired by the source meter.

Supplementary Note 5: Electromechanical response of the sensing elements to cyclic deformations

To evaluate the reliability and durability of our sensing elements, a sample with initial conductance G_0 of 900 nS was fabricated, and a series of bending-unbending recycle tests were then performed. For doing this, a home-made apparatus was used to test the electromechanical performance of the sample under cyclic deformations. Both ends of the sample were clamped by two motor-controlled sliders (see Supplementary Figure 8a). The sample was subjected to bending-unbending cycles when the two sliders, driven by a stepper motor, moved back and forth. The stepper motor was programmed to drive the sliders reciprocating motions for about 8 h corresponding to more than 500 deformation cycles. The sample exhibits a reproducible and stable response with about 7.3% relative change in conductance during the whole cycling test (see Supplementary Figure 8b). The baseline of the response curve began to decrease gradually and slightly after 3 h, which may be caused by the fluctuation of temperature. Taking the insight into the response curve at initial and

ending stages of the long-term test, the sample exhibits consistent and repeatable responses to the deformation cycles without signal recession.

Supplementary Note 6: Mechanical behavior of the PET membrane

Tensile stress-strain test for PET membrane was carried out by using HANDPI HP-100N tensile testing machine. Supplementary Figure 9 displays the stress-strain curve of PET membrane, from which we can see that the values of Elastic limit and Young's Modulus are 1.75% and 2800 MPa, respectively.

Supplementary Note 7: The calculation of altitude pressure

In the barometric altimeter experiment, $\Delta G/G_0$ at each floor can also be calculated from the $\Delta G/G_0$ versus pressure relationship presented in Fig. 2a, and used to compare with the experimental ones. To do such calculation, a pressure verse height curve was firstly calculated from the polytropic atmosphere model (See: website of the National Oceanic and Atmospheric Administration (NOAA) (https://www.weather.gov/media/epz/wxcalc/pressureAltitude.pdf)) with the following formula,

$$P = 101325 \times \sqrt[0.190284]{1 - \frac{h}{0.3048 \times 145366.45}}$$

where P is the barometric pressure at altitude h.

In the calculation, a local altitude of about 25 m (data from Google Earth) was used. In Supplementary Figure 11, the calculated pressure (relative to the pressure at 14th floor) at different height (relative to the altitude of 14th floor) was shown. It can be seen the calculated pressure exhibited a good linearity with the altitude in a small height range. From Supplementary Figure

11, by applying the relationship between $\Delta G/G_0$ and pressure that given in Fig. 2a, $\Delta G/G_0$ corresponding to each floor could be calculated. The results were plotted in Fig. 4c, together with the measured $\Delta G/G_0$ versus height curves. In the calculation, we assumed the height of each floor was 3.5 m. Please note, for convenience the conductance measured at 14th floor was used as G_0 in Fig. 4c. In the barometric altimeter experiment, the barometric pressure at 14th floor was about 500 Pa higher than the pressure of the reference cavity. This means there was a strain existed in the PET membrane when measuring G_0 in Fig. 4c, or say G_0 used in Fig. 4c corresponds to the conductance at 500 Pa pressure in Fig. 2a. Therefore, in the above calculation, when $\Delta G/G_0$ was read from Fig. 2a according to the calculated pressure, a 500 Pa shift on the pressure had to be considered. Fortunately, the abrupt slope change around 60 Pa in Fig. 2a was skipped, which more or less reduced the influence from pressure fluctuations and made the curves in Fig. 4c smooth.

Supplementary Note 8: Electron transport property of the Pd NP array

The investigation of electron transport property of Pd NP array was carried out by measuring current-voltage (*I*-*V*) curves at a temperature range from 20 to 360 K. For variable-temperature measurements, we used a closed cycle cryostat (Janis CCS450) with a temperature controller (Cryogenic Model 32B). The *I*-*V* curves were measured using a source meter (Keithley 2601B).

Supplementary Figure 12 shows typical *I*-*V* curves of Pd NP array. The obtained *I*-*V* curves are highly symmetric and almost linear. The *I*-*V* curves showed distinct deviations at different temperatures. A significant increase in conductance was observed with the increase of temperature leading to the negative temperature coefficient of resistance, indicating that the charge transport of the NP array is highly different from traditional metallic materials. In fact, the charge transport

is dominated by tunneling and/or hopping, enabling this material to be suitable for sensing applications, since the tunnel coupling has an intrinsically exponential dependence on the inter-particle distance which is altered under the deformation of substrates.

Supplementary Note 9: Hysteresis in sensors

We studied the hysteresis of the device with a 0.05 mm-thick PET membrane. We stepwisely increased the pressure applied to the sensor up to 1 kPa and then stepwisely released the pressure down to zero, meanwhile the conductance corresponding to each step was recorded. The results were shown in Supplementary Figure 10a. It could be seen the hysteresis induced by a 1 kPa applied pressure was not significant. The relative conductance showed a shift of 0.012% from the initial value after the pressure loading-releasing cycle. This shift was equivalent to a conductance deviation induced by an applied pressure of 0.9 Pa, which is comparable to the resolution of the sensors. In Supplementary Figure 10b, the largest applied pressure in the hysteresis test was increase to 100 kPa. The PET membrane underwent a plastic deformation, which induced a significant hysteresis.

Supplementary Note 10: FEA simulation of the strain on the diaphragm

To determine the characteristics of the pressure sensor, we performed FEA using the commercial software ANSYS 19. The flexible PET substrate used in the pressure sensor was modelled as a disk with actual geometrical dimensions of 50 μm thickness and 5 mm diameter. A Young's modulus of 2.8 GPa and a Poisson ratio of 0.38 were assigned to the PET. The edge of the disk was fixed. Supplementary Figure 7 shows the evolution of strain on the upper surface of the PET

membrane, where the NPs deposited, under different pressures. It should be noted that the strain at 0 Pa came from the extrusion of clamp on the edge of the diaphragm.

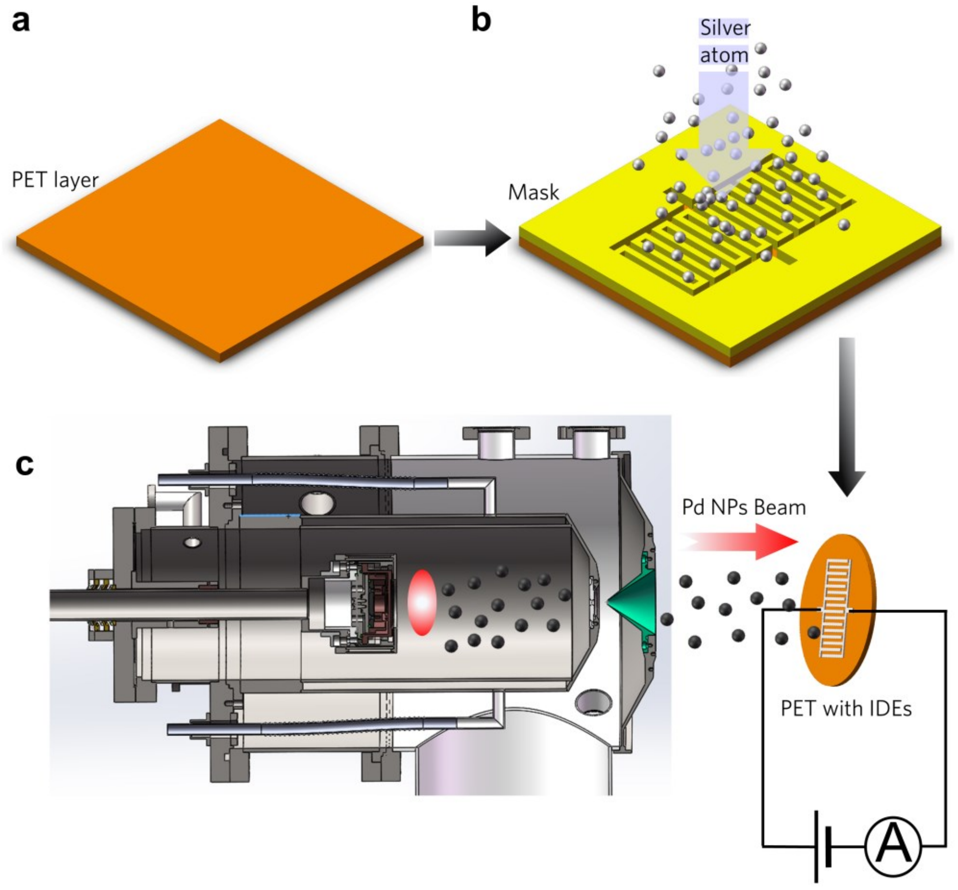

Supplementary Figure 1. Fabrication of actuation layers. a Preparation of the PET layer. **b** Printing the IDEs on PET layers by shadow mask. **c** Deposition of the nanoparticles into the electrodes by real-time monitoring.

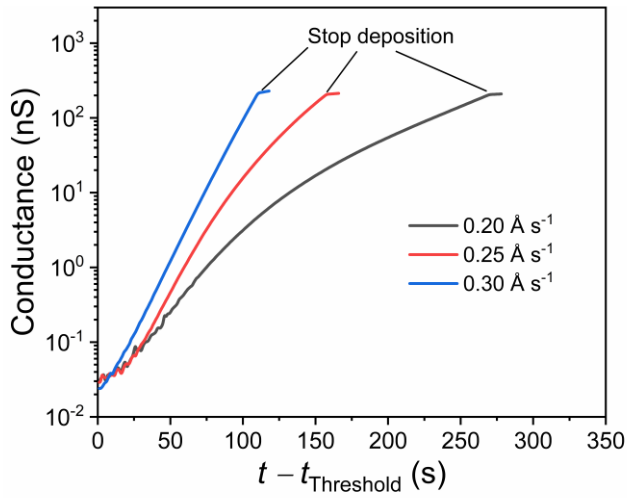

Supplementary Figure 2. Evolution of the NP arrays conductance at different deposition rates. t and $t_{Threshold}$ denote the deposition time and the time when the percolation threshold is reached, respectively. Source data are provided as a Source Data file.

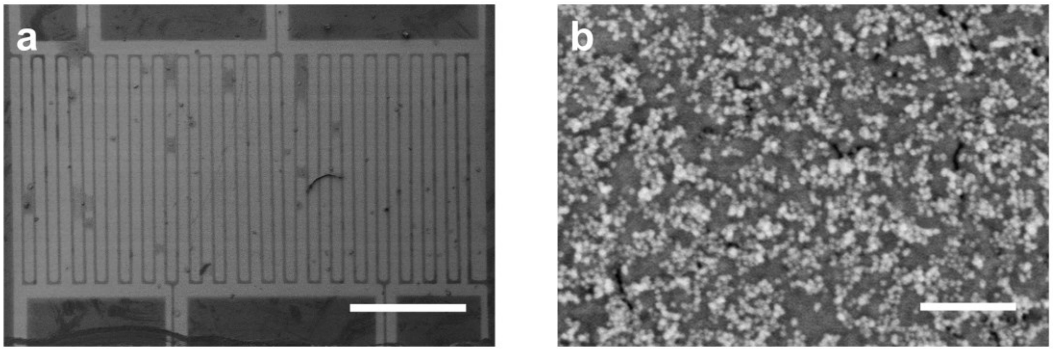

Supplementary Figure 3. SEM images of the pressure sensing element. A low **a** and a high **b** magnifications show the morphology of IDEs and nanoparticle array, respectively. Scale bars represent 1 mm in **a** and 200 nm in **b**, respectively.

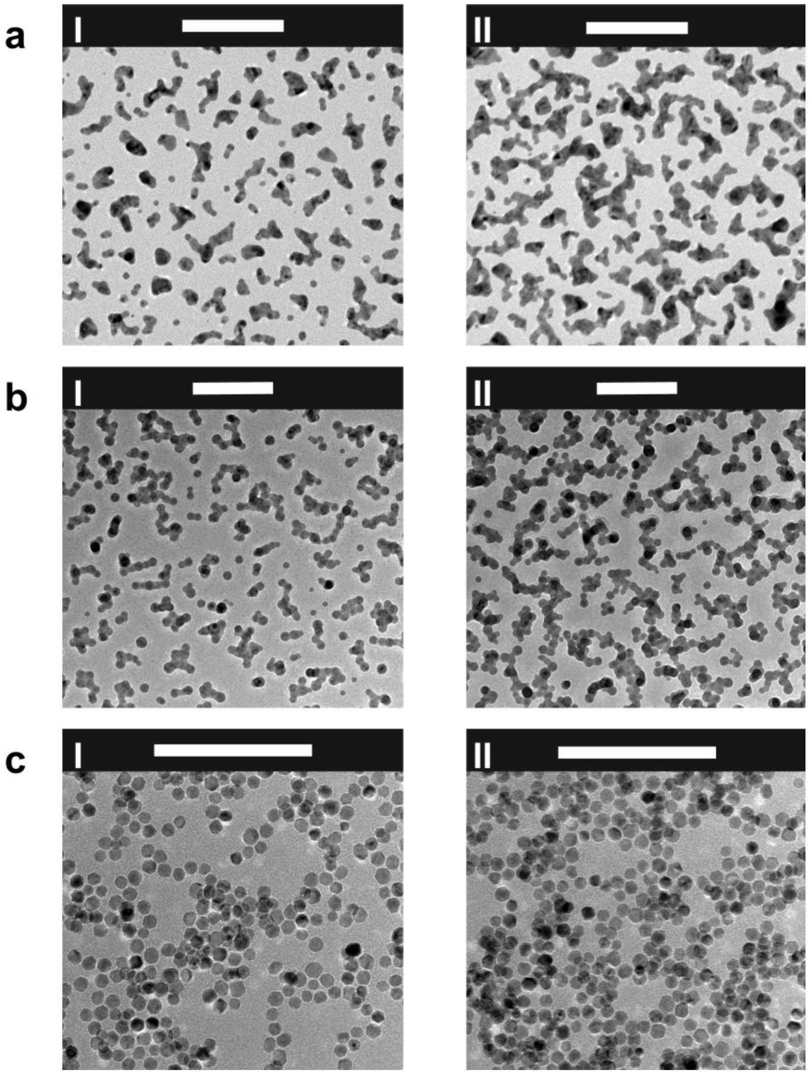

Supplementary Figure 4. TEM images of different metallic NP arrays with different coverages. a Au, **b** Ag and **c** Pd NP arrays with **I** lower and **II** higher coverages are compared. The scale bars in all images are 100 nm.

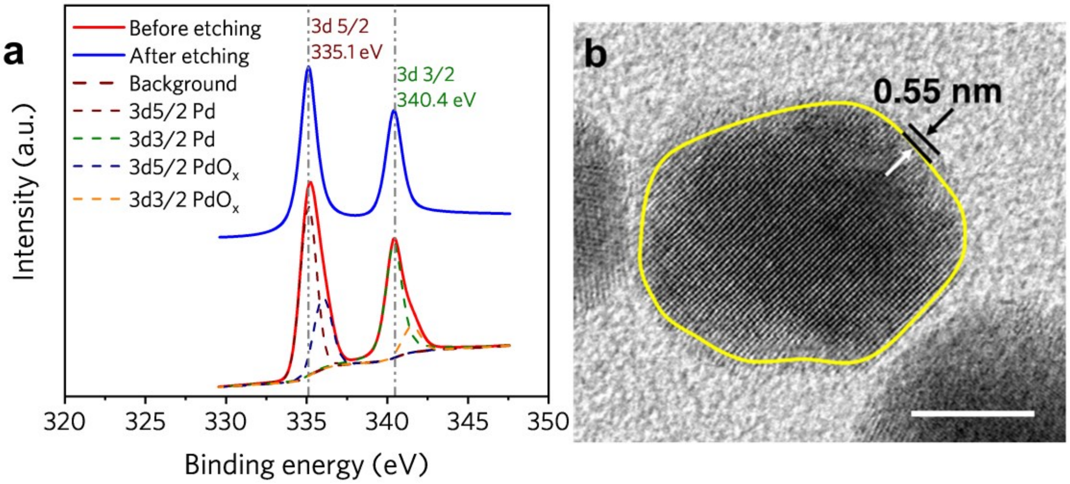

Supplementary Figure 5. XPS and HR-TEM characterizations of the Pd NPs aged for 30 days. a The XPS spectrums of Pd NPs array in the regime from 320 to 350 eV. The accelerated argon iron flow was used to etch off the surface about 3 nm thickness. **b** A typical HR-TEM image of Pd NPs. An extremely thin amorphous oxide layer on the surface of the NP was observed, and its thickness is about 0.55 nm. The scale bar represents 5 nm. Source data are provided as a Source Data file.

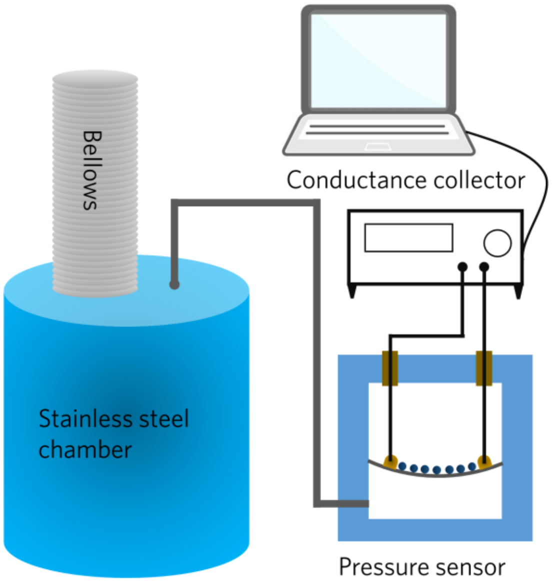

Supplementary Figure 6. Schematic diagram of pressure sensing measurements. This home-made system is used to measure the conductance variation of the pressure sensor under different pressure.

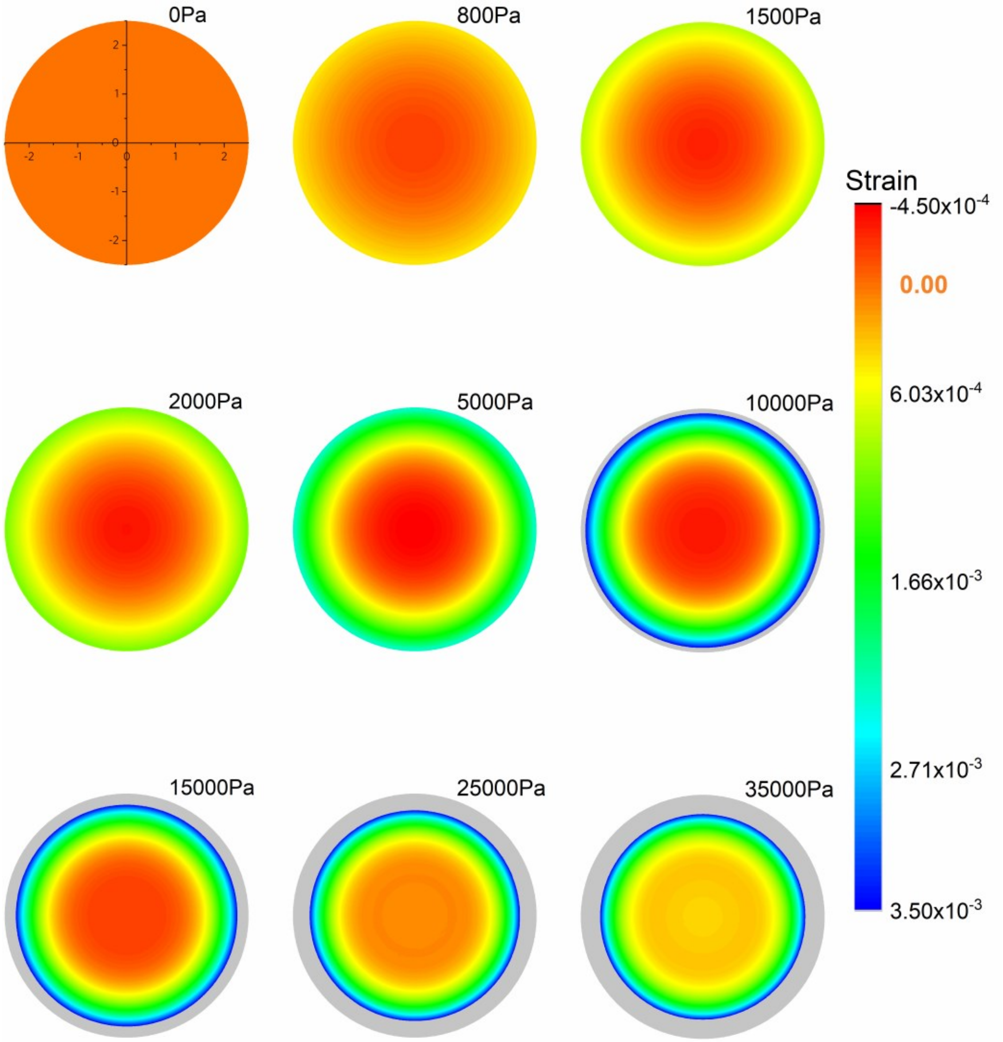

Supplementary Figure 7. The evolution of strain on PET substrate under different pressures. Finite element analysis modeling shows that there is a transition from compressive strains to tensile strains with the increase of the pressure. Source data are provided as a Source Data file.

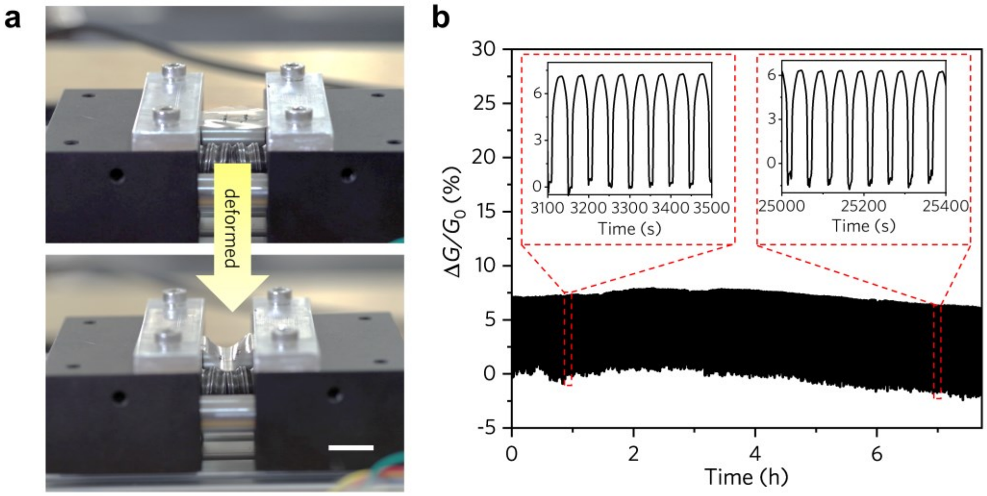

Supplementary Figure 8. Electromechanical response of the sensing element under cyclic deformations. a Photographs of the test equipment. The scale bar represents 1 cm. **b** Relative conductance variations of NP array coated PET membrane under more than 500 deformation cycles. The two insets show some typical cycles at the initial and ending stages of the testing process, respectively. Source data are provided as a Source Data file.

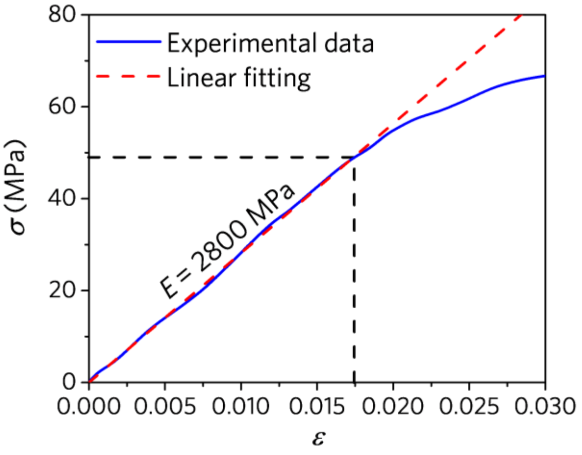

Supplementary Figure 9. Tensile stress-strain curve of the PET membrane. Source data are provided as a Source Data file.

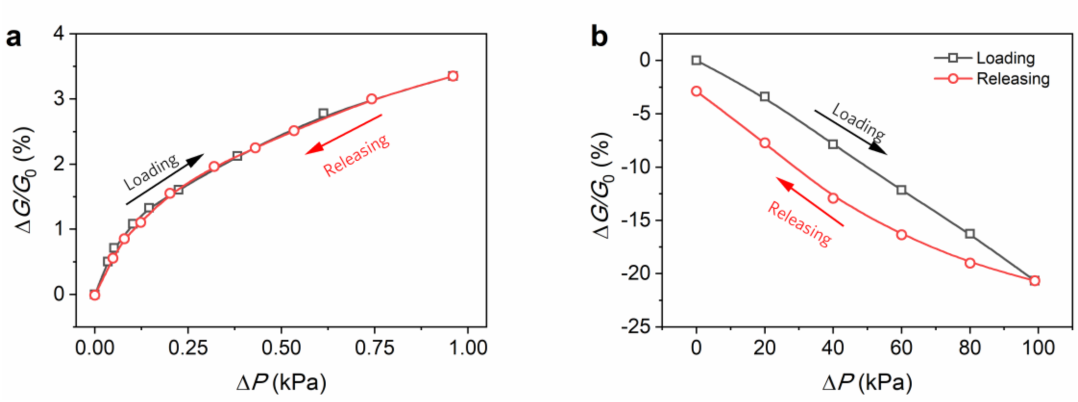

Supplementary Figure 10. Hysteresis measurement. Relative conductance changes of a sensor with a 0.05 mm-thick PET membrane subjecting to stepwisely changed pressure loading-releasing cycles within the pressure ranges of **a** 0-1 kPa and **b** 0-100 kPa. Source data are provided as a Source Data file.

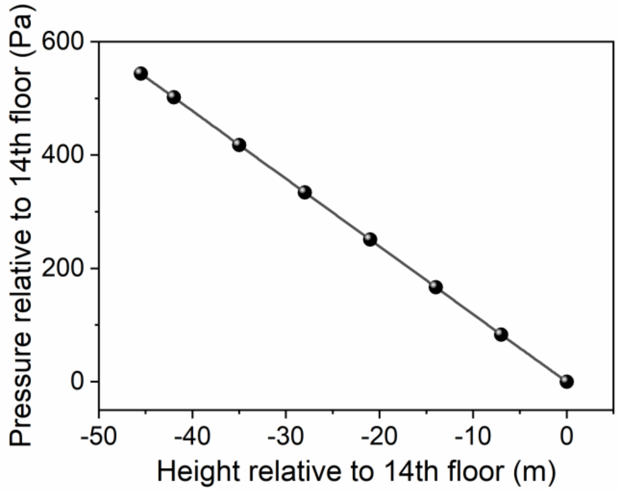

Supplementary Figure 11. A pressure verse height curve. This curve is calculated based on a polytropic atmosphere model. Source data are provided as a Source Data file.

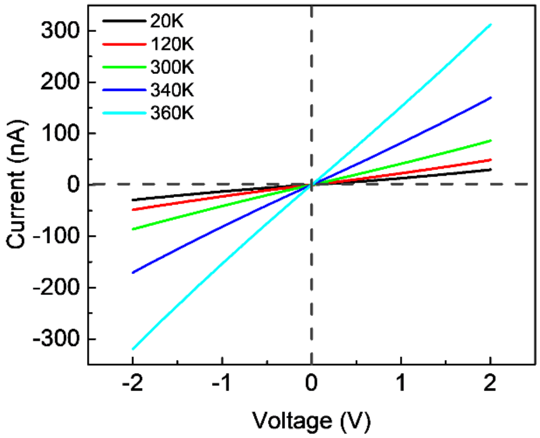

Supplementary Figure 12. Current-Voltage characteristics of NP array at different temperatures. Source data are provided as a Source Data file.

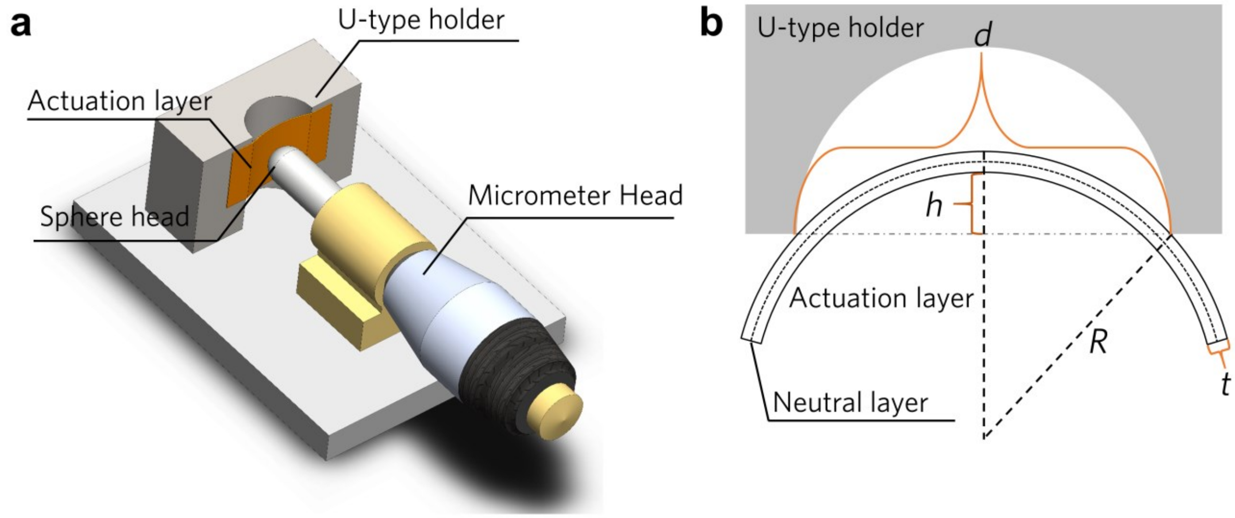

Supplementary Figure 13. Schematic diagrams of strain sensing measurements. a Measurement configuration of the strain sensing test. **b** Geometrical relationship used to calculate the strain. The dash line corresponds to the neutral plane of the PET membrane.

**Supplementary Table 1. Main operating parameters for Pd NPs deposition**	
**Parameters**	**Values**
Aggregation tube pressure (Pa)	80
Discharging power (W)	27, 32, and 37
Deposition rate (Å m^{-1})	0.20, 0.25, and 0.30

Supplementary References

1. Han M., et al. Controllable synthesis of two-dimensional metal nanoparticle arrays with oriented size and number density gradients. *Adv. Mater.* **19**, 2979-2983 (2007).

2. de Heer W. A. The physics of simple metal clusters: experimental aspects and simple models. *Rev. Mod. Phys.* **65**, 611-676 (1993).

3. Wegner K., Piseri P., Tafreshi H. V. & Milani P. Cluster beam deposition: a tool for nanoscale science and technology. *J. Phys. D-Appl. Phys.* **39**, R439-R459 (2006).

4. Palmer R. E., Pratontep S. & Boyen H. G. Nanostructured surfaces from size-selected clusters. *Nat. Mater.* **2**, 443-448 (2003).

5. Yin J., et al. Molecularly mediated thin film assembly of nanoparticles on flexible devices: electrical conductivity versus device strains in different gas/vapor environment. *ACS Nano* **5**, 6516-6526 (2011).
