# Supplementary material for: An ultrahigh resolution pressure sensor based on percolative metal nanoparticle arrays
Source: Nat Commun. 2019 Sep 6;10:4024. doi: 10.1038/s41467-019-12030-x (PMC6731318; doi:10.1038/s41467-019-12030-x)
Supplement: Supplementary file 3 — Description of Additional Supplementary Files [file 41467_2019_12030_MOESM3_ESM.pdf]

Description of Additional Supplementary Files

File name: Supplementary Movie 1
Description: Pressure sensors applied as barometric altimeters. As the elevator rises and falls, the conductance of the sensor displays corresponding responses immediately, demonstrating that our pressure sensors are applicable for barometric altimeters.
